# The Pathology and Treatment of Yellow Fever; with Some Remarks upon the Nature of Its Cause and Its Prevention

**Published:** 1881-10

**Authors:** H. D. Schmidt

**Affiliations:** New Orleans, La.


### ARTICLE IV.

### THE PATHOLOGY AND TREATMENT OF YELLOW FEVER; WITH SOME REMARKS UPON THE NATURE OF ITS CAUSE AND ITS PREVENTION. By H. D. Schmidt, M.D., New Orleans, La.

(Continued from page 312, September No., 1881.)

In drawing a final conclusion from what has been said in the preceding pages upon the bacteria question, we may well presume that they do not represent the direct cause of those diseases with which they have been found associated, unless stronger and more reliable proofs are brought forward in favor of this theory. But, as by this time, in most diseases, the blood and tissues have been examined over and over again by numerous observers, who are anxious, besides, to "find something," without the discovery of other facts than those known for a number of years, it appears quite improbable that this will ever occur.

In order to show that all statements, regarding the presence of minute organisms in the blood and tissues of individuals affected by infectious diseases, must be received with great caution, I shall, before dismissing the theory of the "contagium vivum" in general, cite the views of a few of the most pronounced germ-theorists on the subject. Thus, *Liebermeister*, who theorizes even upon invisible germs, says, more than six years ago:* "It is true that the triumph of the theory of a contagium vivum is in no way complete, and as in former times, so now, it is not so much its opponents, as it is the imprudent adherents who threaten to bring the theory into discredit. The utter lack of critical discernment and method which have characterized some of the works in this field, and, on the other hand, the recklessness with which facts of uncertain significance have been proclaimed certain proofs, have also in our time driven away many earnest investigators."

Koch,† the discoverer of the spores of Bacillus anthracis, after testifying that "in the blood and tissues of the healthy animal or human organism bacteria are not met with," continues as follows: "It appears to me, however, that the following objections against the acceptation of bacteria being the cause of infectious traumatic diseases are justified. To gain an incontestable proof for this acceptation it is essential *that the bacteria, without exception and in proportions regarding their number and distribution, should be shown in such a manner, as to perfectly explain the symptoms of the respective disease.* For, if in some cases of a certain kind of infectious traumatic disease bacteria are found, while they are absent in other cases exactly alike,—and if, furthermore, the bacteria are present in too small a number as possibly to cause a severe disease or even a fatal issue, it becomes self-evident that their inconstant appearance is dependent upon chance, and their small number no sufficient proof for regarding them as the sole cause of the respective disease, and that we must accept the existence of other causes besides. The observations made in regard to the presence of bacteria in infectious traumatic diseases, however, do in fact not correspond with the demands required for an incontestable proof."

* Cyclopædia of the Practice of Medicine, edited by Dr. H. v. Ziemssen; American edition, Vol. I.

† Koch, R.—Untersuchungen ueber die Ætiologie der Wundinfections-krankheiten. Leipzig, 1878, p. 22.

*Birch-Hirschfeld*,* in speaking of the pathological signification of bacteria makes the following remarks: "But it must be admitted that, even if the constant occurrence of these organisms in the diseases mentioned is recognized as a fact, doubts may yet arise as to whether these bacteria are the real cause of the disease."

"This question will not arise with an infectious disease in which organisms of a certain morphological character constantly and exclusively occur, as in recurrent fever, where the spirilla claim a diagnostic signification. In splenic fever this circumstance is less significant."

"The morphological character of the bacteria found in pyæmia, diphtheritis, small-pox, cholera, however, are so much the same, as to give rise to the idea that they are identical forms. But the conclusion drawn from this would be that no specific signification could be assigned to these organisms. Accordingly, they would not represent the cause but parasites of the disease, which, even then, may not be without signification, or may stand in some relationship with certain septic conditions of some infectious diseases."

After the preceding discussion of the so-called "bacteria theory" and of those diseases in which these minute organisms are really met with in the blood and tissues of many cases, we are now ready to inquire *whether this theory may in any way be applicable to yellow fever;* and, in order to arrive at correct conclusions from such an inquiry, it becomes essential that we should set aside all postulation, such as the existence of "invisible" living germs, and strictly keep to observed positive or negative facts. It is self-evident, therefore, that the assertion of bacteria or their spores being the cause of yellow fever must be substantiated by the ocular demonstrations of these organisms in the fresh blood of the patient, before it can be received as a truth. Judging from my own numerous examinations of yellow fever blood under various conditions, already mentioned in this treatise, I need not hesitate to say that this has been done. And, since the results which I repeatedly obtained ever since the yellow fever epidemic of 1867, have now been corroborated by those

* Birch-Hirschfeld.—Lehrbuch der Pathologischen Anatomie, p. 233.

of *Sternberg*, who, during 1879, examined quite a number of specimens of fresh yellow fever blood at Havana, I may add that all statements to the contrary must have been founded upon an imperfect and inexact manner of microscopical examination. And as, furthermore, bacteria are neither met with in the tissues and organs of fatal cases of yellow fever after death, if properly prepared while still in a fresh condition, we are justified in presuming without further extending the argument, *that yellow fever does not depend upon the presence of bacteria or any other minute living organisms in the blood or tissues*, for, if it did, *the microscope would most certainly show them*, as well as it has done in those other particular diseases mentioned.

But, it will now be asked, if the cause of yellow fever is not represented by minute living organisms, what is it then? And there are many persons, both physicians and laymen, who will promptly answer this question by referring the cause to the products of vegetable and animal decomposition in the form of filth and dirt in the streets and houses. But before I shall attempt to show that the "filth-theory" has no better foundation than the "living-germ-theory," it becomes necessary that I should properly define the standpoint from which I view the subject, as otherwise I might be looked upon as an advocate of dirt and filth. There is hardly a physician who does not consider a supply of fresh and pure air and water as the cardinal objects in public and private hygiene; nor have I, myself, ever regarded these elements in any other light. On the contrary, the degree of civilization of a community may rightly be judged from the degree of neatness and cleanliness of the houses and streets; and nobody will doubt but that the hygienic condition of a community depends to a considerable extent upon the purity of the air breathed by the people in their dwelling-places. It is also true that some local affections may arise from the breathing of air contaminated by gases arising from privies, or from other animal and vegetable decomposition. Such cases may occur in any large city, though not very often; but, that a *specific* and *contagious* disease, characterized by *specific symptoms and pathological changes in the tissues and organs*, should take its origin from such a cause is contrary to all modern scientific knowledge. A

specific disease can only be caused by a specific poison; and though certain pathological processes may equally take place in different specific diseases, yet each of the latter maintains its own specific character besides. The poison of yellow fever will never produce scarlatina, and the poison of the latter will never give rise to yellow fever, though in both diseases parenchymatous degeneration of the kidneys may be met with. The development of a specific disease may be compared with that of the egg; for a certain mysterious agency—similar to that determining the specific characters of an animal or plant, and lying dormant in the protoplasm of the egg-cell—also resides in the poison of a specific disease, determining the specificity of its symptoms and pathological changes. And, accordingly, the inquiry into the origin of these specific poisons will be as resultless as that into the origin of the first egg. As far as I am able to judge, it appears to me that specific diseases have never sprung suddenly into existence, but that their origin must be compared to that of vegetable and animal species, and that, throughout an immense length of time, they have been developed into what they are now by the processes of "selection" and "adaptation." Thus, small-pox, or yellow fever, may have presented a different character thousands of years ago, from what they do at present.

Yellow fever, therefore, being a specific disease, cannot arise spontaneously through the influence of the air rendered impure by the products of vegetable and animal decomposition, for, if this were true, New Orleans would be depopulated long ago. From all I know, the disease, in this city, has never arisen at the outskirts, or in the vicinity of those places to which the kitchen refuse and rubbish of the houses used to be carried not many years ago, or of grave-yards, such as the Locust Grove Cemetery of bad repute, but, on the contrary, always in the populated commercial centers, or in the vicinity of the harbor. Besides, it is a historical fact that this disease almost always first appears in a sea-port, before it extends into the interior of the country, and that it stands in close relationship with the shipping over sea. If yellow fever could ever have originated from the gases arising from decomposing matter, it should have certainly done so in the vicinity of one of those so-called dumping grounds, a large empty lot, situated, not many years ago, on Broad Street Canal between St. Anne and Dumaine Street, and which, in the pursuit of my practice, I had frequent occasion to pass. Whenever I passed, I found it occupied by quite a number of negro rag-pickers of both sexes, accompanied by their dogs, and engaged in digging the ground in search of lost treasures. The stench arising from this place was so offensive and sickening in character that I had to rouse my horse and hold my breath until I had passed the whole square. In the same way I have frequently met with offensive odors, though differing in character from that of the garbage, arising from the stagnant water of the canal itself, yet I never heard of yellow fever originating in that vicinity, nor did I learn that other diseases prevailed to a greater extent there than elsewhere. As regards the spontaneous origin of diseases in the vicinity of the grave-yards in the city, I need only point to the report of a committee, appointed by the Medical and Surgical Association of New Orleans, for the investigation of this matter, and which answered the question in the negative. Many other instances might be adduced to show that no relation exists between the origin of yellow fever and the filthy condition of some of the streets of New Orleans, or any other city. With this assertion, however, I by no means advocate the existence of grave-yards in the heart of the city, or bad drainage and muddy and filthy streets and houses, for there remains no doubt but that whenever the the disease has once been introduced and assumed the character of an epidemic, nothing will contribute more to it being fostered and propagated than filth and insufficient ventilation. This I know from experience.

It has also been asserted that yellow fever was caused by gases arising from the bilge-water in the hold of the ships, and the latter has even been regarded by some germ theorists as the medium by which the "contagium vivum" was transmitted from port to port. As regards the gases emitted from foul bilge-water, the remarks made on filth and dirt will be equally applicable to this liquid; but, as concerns the living germs which it is supposed to contain, the supporters of such a theory seem to have forgotten that these minute organisms are all inhabitants of the water or other liquids, which they are incapable of leaving except by evaporation; for, minute as they are, they are still too heavy to be lifted out of the liquid with the molecules constituting its vapors, while in a dry condition they may be carried about by the slightest current of air.

Besides the reasons I have given above for showing the futility of the theory of yellow fever originating in filth, or from the gaseous products of decomposing matter, I might still further enlarge upon this subject,—a subject which, by having been treated so often and so extensively, both in medical and public literature, is now too well known to require any further comment on my part. Nevertheless, in support of my own views, I cannot forbear to cite the views of two distinguished germ-theorists before dismissing it. Thus, Liebermeister* says: "Not a very long time ago it was almost universally accepted that merely the coincidence of certain especial conditions were necessary to cause the autochthonous appearance of certain infectious diseases. As early as the time of the Athenian plague, Diodorus found a sufficient explanation for the origin of the disease in the circumstance that a great multitude of people from all quarters streamed into the city, and, being cramped for room, breathed a corrupted air; "thus they were attacked with disease in an inexplicable manner." How many thousand times since then have medical and non-medical writers pictured social squalor, decomposing filth, unfavorable weather, etc., as the cause of disease! And physicians and laymen were accustomed to take it for granted that the plague and similar severe epidemics originated after this fashion. Many physicians found no difficulty in explaining the origin of syphilis from the mingling and concentration of the immoral elements of numerous nations. The plague arose from the imperfect burial of human corpses, with the consequent corruption of the air; yellow fever originated from foul bilge-water or from crowding of men together in slave ships; typhus fever from the crowding together of persons in badly ventilated dwellings, or from hunger; the cholera from rotten or unripe vegetable food; the typhoid fever from the exhalations of putrid excrements, etc. For many it was an interesting spectacle to realize

---

* *v. Ziemssen*, "Cyclopædia of Practice of Medicine," Vol. I, p. 17.

how the great regulatory operations of nature should be so simple and easily understood, and how every considerable deviation from a proper observation of hygienic laws should immediately be punished by the production of a particular disease. In recent times the standpoint has been essentially changed. The potency of the factors in the *extension* of the diseases in question is not questioned; on the contrary, our knowledge of it has become more reliable and exact. We have learned, however, that the diseases do not *originate* in this way. It was observed that the battle-fields of Inkermann, strewed with corpses, whose stench drove the armies away, produced no pestilence. We have been convinced that, in spite of the prophecies and premature results, the investment of Metz was never able to produce a single case of typhus fever, inside or outside of the city, or to transform typhoid fever, which prevails there so frequently, to a possibly higher potency, *i. e.*, to typhus fever. We have gradually reached the conclusion that it is only where the specific germ of the disease exists by itself, or has been introduced, that those anti-hygienic factors become active and may then be capable of occasioning an enormous extension of the disease. The germ, however, is not produced by spontaneous generation."

"In fact, at the present time the acceptance of an autochthonous origin for syphilis is, for most physicians, as great an absurdity even as the origin of scabies or of lumbricoid worms by spontaneous generation. It is unanimously said of the plague, cholera, and yellow fever, that they never arise spontaneously, at least on European soil. The spontaneous origin of small-pox, measles and scarlet fever could hardly find a defender now. Perhaps the time is not far distant, when the doctrine of the spontaneous origin of typhoid fever, dysentery, typhus fever, etc., will be universally rejected."

Naegeli, from whose work on "The Lower Fungi and Their Relation to Infectious Diseases," I have already quoted in this section, speaks of the influence of the air upon these diseases as follows: "The air, containing bad smelling or other gases, though disagreeable, is not rendered noxious by it, particularly with regard to infectious diseases. With it, however, the different forms of more or less dangerous cleft-fungi, suspended in the dust-like masses, may be introduced into the body."

"The infected air itself is odorless. A putrefying substance only becomes dangerous after it has become dry, and the bad odor has disappeared. The miasms and contagia are also imperceptible for the organ of smell."

"The microscopical and chemical examination of the residue of filtered air teaches us nothing as regards its noxiousness, as the infectious matters cannot be distinguished. It will be impossible to pass a judgment upon the infectious nature of any air, before we are able to test the effects of this residue in an experimental manner."

"It is impossible to render infected air innoxious, as it cannot be made free from dust. At the utmost, the dust suspended in the air of an inclosed space may be removed by the vapor of water, or by sprinkling the walls."

By some the origin of yellow fever, as well as that of other diseases, has even been referred to our drinking water becoming pregnant with decomposing vegetable matter while standing in the over-ground cisterns. I shall once more draw from Naegeli's work and cite this author's opinion on this subject. He says: "The drinking water (from wells, rivers, ponds, lakes, ground-water), even if not clean, is not deleterious to health (provided poisons do not get accidentally into it), and, least of all, causes infectious diseases, as those substances rendering it unclean are generally harmless in their nature; the fungi of putrefaction and putrid substances, as well as miasma-fungi, which it contains, represent so small a quantity, as to reduce the probability of a contagion to an imperceptible minimum."

"With this corresponds the experience, which shows that the continual eating of large quantities of humus substances and products of putrefaction, as are contained in foul drinking water, as well as the exclusive and constant use of the latter by whole populations, are without deleterious hygienic effects. A critical consideration of the known facts relating to the spread of infectious diseases, also, shows that contagion has been wrongly ascribed to the water."

The idea that the air we breathe is pregnant with fungus spores and bacteria, and that hundreds of these minute organisms are inhaled with every inspiration, seems to prevail among a number of medical men; and it is this coarse error which has rendered the germ-theory so acceptable to the medical practitioner. It is true that under certain conditions these minute organisms are met with in the air, though in a comparatively small number, for the reason that the natural media in which they live are fluids in a certain state of decomposition, in which they multiply to countless numbers. Their comparative scarcity in the air has been satisfactorily proved by the observations of *Cunningham*, made some years ago at Calcutta, for the purpose of determining whether there existed any definite relation between the quantity of organic matter in the air and the prevailing diseases, such as diarrhœa, dysentery, cholera, miasmatic fever and dengue, or whether these diseases depended upon certain forms of organisms suspended in the surrounding air. For this purpose he used a weather-vane in the form of a tube, containing a plate of glass covered with a layer of glycerine, to which all particles contained in the surrounding air would adhere. The apparatus was placed in the vicinity of a prison and five feet above the ground. The matter adhering to the glycerine consisted of particles of silica, amorphous granules, carbon, lime, starch granules, cells, hair, fragments of vegetable tissue, filaments of cotton, hairs of insects, oil globules, pollen granules, spores and cells of fungi and algae, sometimes also bacteria, but in a small number. It was impossible to show a relationship existing between the amount of these particles and the diseases above mentioned.

In New Orleans, where rain-water, collected from the roofs of the houses into over-ground cisterns, is used for drinking or cooking purposes, similar objects are frequently found in the water, especially after a long draught, when the dust floating in the air has had sufficient time to settle upon the roofs of the houses. But I have never heard that diseases have arisen from the drinking of the cistern-water containing these miscellaneous particles, except, perhaps, when the water had been standing for a long time in an old foul cistern,—and even then such an occurrence would be very improbable. On the contrary, our drinking water is the purest that could be anywhere obtained, especially after being filtered, as it is in reality distilled water, in the place of which it is also generally used by the apothecaries. I have been using this water now for many years in my microscopical examinations, but I do not remember ever to have met with bacteria in it, though, if used unfiltered, minute fragments of vegetable tissue or other particles may be met with. Bacteria do not appear until putrefaction has set in.

A number of times I have had to listen to the remarks of medical friends that living germs might exist in the air, too minute to be seen by the aid of the microscope. To this I can only reply that the modern "germ-theory" has not been based upon "invisible" germs. Even Hallier and Pasteur, the most persevering germ-theorists, never attempted to substantiate their assertions by things they could not see. They only failed in proving that the germs or organisms which they did see represented the true cause of disease. The smallest organisms known at present are the monads, bacteria and vibrios, representing minute granules, either single or arranged in rows, as already mentioned. But minute as they are, they really require no high amplification to be perceived in the form of a dot; it is only for the study of their details that high magnifying glasses and correct illumination are required. And, as stated before, the modes in which they multiply have even been studied by the aid of the microscope. The modern microscope is fully able to show things much smaller than these organisms, regarded by the germ-theorists as the cause of the disease; and, unstable as the germ-theory may be, it is surely not based upon imaginary objects. Its failure only consists in its inapplicability to the phenomena associated with the pathological processes of the disease in question.

In the preceding pages I have endeavored to demonstrate that the cause of yellow fever cannot be assigned to the noxious influence of bacteria in the air, for the potent reason *that they are not met with in the blood and tissues* of yellow fever patients, either before or after death. Neither can it be assigned to the noxious effects of the breathing of air contaminated by the products of vegetable or animal decomposition, or to foul drinking water, for such a supposition would be, as I have endeavored to show, in opposition to all experience of modern science, teaching that infectious diseases never arise from these last mentioned causes. There is, however, another "invisible" agent left, to the noxious influence and effects of which all the characteristic features of the disease may be referred. This agent represents a certain specific noxious poison, *being a product of the diseased organism itself*, and, in consequence, *of animal origin.* To show the probabilities of this supposition, and to explain the more than probable manner in which this poison is reproduced and increased in quantity, as well as transmitted from individual to individual, will be our next task.

When, in 1867, the yellow fever appeared again at New Orleans, and soon extended over the city in the form of an epidemic, I adopted the view then generally entertained by the larger portion of the medical profession and the people of this city, that the disease was non-contagious; and as the fungi-theory, originating in the statements of Klob and Hallier, was then prevailing among medical men, I also inclined to the view that yellow fever depended upon some kind of micrococcus floating in the air. And, in accordance with this idea, I examined the mucous membrane of the alimentary canal and respiratory passages in quite a number of cases at the Charity Hospital, but without discovering any micrococci or other minute organisms in those localities; in the same way I failed to discover anything abnormal in several specimens of blood from hæmorrhages of the nose occurring in some of my private patients, or from specimens taken from the dead body at the Charity Hospital. It was only in the black vomit, as already mentioned, that I met with fungi and their spores. But as it was obvious that the organisms contained in this fluid could not have any relationship with the primary cause of the disease, I did not hesitate to discard the fungi-theory, while retaining the view of the non-contagiousness of the disease. And, in view of the analogy certainly existing between some of the symptoms of miasmatic and yellow fever, I became inclined to regard the latter as a miasmatic disease in the most intense degree, depending upon a poison arising from a combination of decomposing vegetable and animal matters. A reason for this view was that the disease principally prevailed in cities in which large numbers of men and animals lived in close proximity along the borders of swampy rivers. This view, which is still entertained by a number of physicians, I favored until the yellow fever epidemic of 1878 made its appearance.

Previous to this time my studies had been principally directed to the pathology and treatment of the disease; and, I must confess that, since I failed to demonstrate the primary cause of yellow fever upon the theory of a *contagium vivum*, I had paid but little attention to this subject afterward. But when I observed, during this last epidemic, the disease slowly extending, frequently from house to house—and, in many instances, after having once entered a house or a family, not leaving it until it had attacked every inmate who had not been affected by it before,—when I saw it traveling from one district of the city to another, new centers of infection arising in places to which the poison had evidently been carried; and when the epidemic slowly extended from our city to neighboring communities—especially along the railways and other routes of public travel,—even to villages and plantations in the country, my attention was again directed to its cause, and by subsequent and more systematic studies and observations I became convinced of its contagious nature.

It would be impracticable to enlarge upon this subject in this place by citing and discussing individually the numerous cases and instances which speak in favor of the contagiousness of this disease, I therefore only refer the reader to the innumerable reports, both of ancient and recent date, containing the history and statistics of yellow fever. I may, however, remark that any physician who will take the trouble and devote the time to an impartial and philosophical study of the subject in all its details, following the outlines which I have sketched in this treatise, will finally become convinced, like myself, of the contagious nature of this disease. My own observations and studies have even inclined me to regard the infectious poison of the latter as more subtile and virulent in its nature than that producing small-pox.

Yellow fever, then, is a disease, running a regular course, and accordingly depending, like small-pox, scarlet fever, measles, and other kindred diseases, upon a specific poison of animal origin, being a product of the diseased human organism itself. This assertion is proved by the characteristic feature which it presents in common with all other specific diseases, consisting in the *immunity* from a second attack, imparted to the individual once affected; and this immunity from a second attack, which most strikingly distinguishes contagious diseases from all others, must be constantly kept in view in the study of the nature of the peculiar poison producing yellow fever.

The precise nature of the particular pathological changes which impart to the organism this immunity from the effects of the poison afterward, we are at present unable to determine. Perhaps the most popular view is that the poison alters the constitution of the blood in such a manner, and so permanently, as to be unable to make any further impression upon it afterward. It is equally difficult to say whether the changes are wrought upon the colored blood corpuscles in particular, or upon the plasmatic portion, the liquor sanguinis furnishing the nutriment to the organs and tissues of the body. Judging from the unusually rapid disturbance in the nutrition of the organism, manifested by the phenomenon of fatty infiltration and degeneration in a number of organs, and by the nervous disorders, the liquid portion of the blood would appear to be first affected; while, on the other hand, the facility with which the blood corpuscles seem to part with their hæmoglobin, as my observations have shown, indicates that these bodies must be likewise affected. For a number of years I have, in relation to the difference existing in the impression of the poison upon the blood in miasmatic and yellow fever, been in the habit of illustrating it in a somewhat homely manner by assuming that in the former the impression of the poison is only functional in character, and in consequence evanescent, while in the latter it is organic in its nature, and therefore permanent, and insuring the immunity from a second attack. But, though it appears to me possible that the immunity may depend upon certain permanent changes wrought by the poison upon the blood—difficult as it may be to explain—I cannot forbear to presume that similar changes may at the same time take place in the nervous tissues, rendering them in the same manner unimpressible for another attack.

Besides the immunity from a second attack, characterizing yellow fever as a disease produced by a specific poison, the clinical symptoms, and, moreover, the anatomical changes observed after death, also speak for its peculiar and specific character. But as a specific disease cannot be produced in the human body by a general cause, it is obvious that the specific poison producing it must be derived from another human body affected by the same poison, and that this poison must, necessarily be a product of the organism of the affected individual. We may, therefore, presume that a *general* cause cannot produce a specific disease.

Yellow fever, when it first appears, never attacks a number of persons isolated from each other at one and the same time, but always, like every other contagious disease, starts from single centers, represented by the infected individuals; and the only manner in which it spreads is either directly from individual to neighboring individual, or indirectly, when the poison, emanating from the affected individual and adhering to clothes and other objects, is carried to a distant place and inhaled by another person. A number of centers may thus arise in different localities of a city, which, increasing in dimensions, may at last meet. In this manner, alone, the infectious poison is able to extend over a large city like New Orleans; and the surest proof of this assertion is offered by the slow march in which the disease walked over our city during the last epidemic. A purely infectious poison, on the contrary, being contained and distributed throughout the air of a city or locality, whether in the form of living organisms, or in the form of vegetable or animal effluvia arising from decomposing matter, will necessarily affect every person susceptible to infection nearly at the same time, and, in consequence, rapidly extend over a city or district. For this reason, the theory of a contagium vivum, or of the miasmatic or effluvial origin of yellow fever, is entirely inconsistent with the contagious character of the disease. But notwithstanding these existing circumstances, there are still a number of physicians who, while they recognize the contagiousness of yellow fever, entertain at the same time the incongruous idea that the cause of the disease is represented by minute living organisms, and for the sole reason, as they say, that this is the only theory upon which they can understand the increase of the poison, as nothing could spontaneously increase unless it were endowed with life.

I shall try to explain this subject by a suitable comparison. In every organized body, whether plant or animal, we meet with an albuminous substance which is regarded as the basis of life, and is generally known by the name of "protoplasm." This protoplasm, as we all know, forms the basis of all tissues performing a vital function, whether consisting of cells or fibers; and though it is not an organized body, it nevertheless represents living matter and is endowed with the power of appropriating other matter unlike itself—such as the nutritive matters derived from the food and contained in the blood,—and of imparting to it its own properties; or, in other words, of converting this matter into protoplasm. Now, it is upon this principle that all tissues and organs of the body not only rejuvenate themselves, but also increase in size. Thus, the protoplasm of a muscular fiber appropriates new matter and converts it into muscular fiber; the protoplasm of a ganglion-cell imparts its properties to the same new matter furnished by the plasma of the blood, and converts it into nervous tissue, etc. The new matter is in all cases the same, but it is the protoplasm of each particular tissue which represents the converting agency.

The specific poison, having entered the circulation, and being diffused throughout the blood, imparts, like the protoplasm, its own properties to this fluid, giving rise to all the pathological phenomena characterizing the disease. Still, it must be remembered, that the degree of intensity of the disease depends entirely upon the quantity of the poison taken up by the blood. If the quantity is sufficiently large to affect all the blood circulating through the body, the disturbance of nutrition would be so great as to interfere at once with all the vital functions, and the case would prove fatal very rapidly. We may, therefore, presume that, with some rare exceptions, in all cases of yellow fever the poison only affects a smaller or large portion of the blood, leaving to this fluid sufficient integrity to nourish the tissues and organs at least for some days, thus affording an opportunity to the organism to rid itself of the poison. This, however, can be accomplished in no other way than by means of the action of secreting, or, as we may say in this case, excreting cells, the same organs through which the organism rids itself of all other foreign or wasted matter. Thus, the poison, already increased in quantity by that portion of the blood to which it imparted its own properties, is absorbed from this fluid by the cells of different glandular organs during its passage through the capillaries surrounding the individual minute glands. And, in passing through these cells, it again increases in quantity *by imparting its noxious properties to their natural secretion*, together with which it is finally eliminated from the organism. In yellow fever therefore, as in all other contagious diseases, it is in the secretions, especially those of the glandular cells of the skin and of the respiratory passages, that the noxious poison is contained, and with which it is removed from the system.

In some contagious diseases, the poison, before leaving the body, gives rise to certain pathological processes in the epidermic cells of the skin, manifested by divers exanthematous eruptions, which, in some instances, as in small-pox or the cow-pox, proceed to the formation of vesicles and pustules containing the poison in liquid form. Nevertheless, even in these affections, a portion of the poison leaves the body in a gaseous or vapor form, possessing the same virulent properties as the liquid contained in the vesicles or pustules. In yellow fever the poison emanates from the affected individual only in a vapor form, in which it may be communicated to another individual, or may adhere to surrounding objects, as clothes, bedding, furniture, etc. In the latter case, however, it may be supposed that while adhering to these objects it undergoes a certain condensation by drying, and in which state it is transported to distant places. But, as soon as acted upon by a certain degree of moisture and heat, it again assumes the vapor form in which it may be communicated to other persons.

In holding the view of the contagium of yellow fever or other contagious diseases being transmitted from one person to another through the vapor arising from the secretions of the skin and lungs, we need not presume that the whole vapor represented the noxious poison ; on the contrary, we may concur with the opinion of Hiller, already cited, namely, that the ferments of putrefaction are represented by most minute proteïn-particles, which, being themselves in a state of putrefaction, are carried through the air to the substratum to involve it in a similar decomposition. In the case of yellow fever, therefore, these proteïn-particles may be derived from the protoplasm of disintegrated epithelial cells of the glands of the skin to which the properties of the specific poison had already been imparted, and which, on their part, if entering the blood of another individual, are also capable of imparting these properties to the constituents of this fluid. This explanation corresponds with the theory that only solid bodies are capable of acting as ferments. Accordingly, the spread of the disease would be affected by the expansion of the vapor of the cutaneous secretion containing these particles into the air surrounding the patient, and inhaled by other individuals—and, furthermore, by the secretion adhering to the clothes and bedding of the patient, or to other neighboring objects; if the perspiration be profuse, as is generally the case, it will also be absorbed by the clothes and bedding.

In yellow fever as in small-pox the secretions of the skin are generally associated, as already remarked, with a peculiar odor. Whether this odor really represents the noxious principle, or whether, being somewhat ammoniacal in character, it is due to the decomposition of urea contained in the secretion, I will not pretend to determine. But, judging from its strength as it emanates from a single patient in a badly ventilated sick-room, we may form an idea of the facility with which the vapor, pregnant with the poisonous principle, may adhere to the clothes of visitors to be carried to distant places, or how easily it may communicate itself through the open windows to neighboring houses and people. But, in order to decide upon the probability of this mode of propagation of the noxious poison of contagious diseases, we should compare it with the manner in which odorous bodies impart their odor to the surrounding air, or to neighboring objects. Although nothing definite is known of the manner in which odors are diffused, or of the nature of the impression which they make upon the ultimate terminations of the olfactory nerves, it is nevertheless supposed (Cloquet),* "that many bodies possess the property of setting free from their substance exceedingly fine

* *v. Vintschgau*,—"Physiologie des Geruchssinnes"—Handbuch der Phisiologie, herausgegeben von Dr. L. Hermann, Vol. III, part 2, p. 161, 1880. (Still under publication.)

particles, forming a kind of atmosphere around the odoriferous body and becoming diffused into the surrounding air; their density diminishes in proportion to their distance from the odoriferous body. Accordingly, the air represents the medium by which odorous substances are spread."

"*Cloquet*, *Bidder*, *Valentin*, *Longet*, and *Poinsot*† have mentioned a number of conditions under which the odor is developed. There are, namely, odoriferous bodies the substance of which is incessantly, either partially or entirely, volatilized, while others only became odoriferous under certain circumstances. There are, for instance, plants which exhale only during daytime, or only during the night, or, even, only in the morning. Some plants emit odor when they are dried; a generally known example of this is found in freshly cut hay. Aromatic herbs possess in the dried condition only a feeble odor, but smell tolerably strong when moistened. Bituminous substances have no odor in the dry condition, but a distinct one when damp. The dampness appears, therefore, to favor the emanation of the odor, and still more, if the evaporation is supported by a moderate temperature; heat destroys the odor, and a low temperature arrests its emanation; these lower limits differ with different substances."

"*Liégeois* arrived at the conclusion that minute particles are constantly diffused from the odoriferous bodies, especially when they are in contact with water, in the atmosphere, and arriving at the Scheiderian membrane with the olfactory organ."

From these citations, then, we find that odors are produced by minute particles emanating from the odoriferous body to become diffused throughout the atmosphere, and their emanation is favored by dampness, especially at a moderate temperature. But there are also bodies of vegetable and animal origin which emit no odor, and yet manifest the same phenomena indicating the emanation of fine particles as from the odoriferous bodies. It is, therefore, not essential that a substance, in order to emit or set free minute particles, should be odoriferous. The tenacity with which odoriferous particles adhere to other bodies, especially such of a loose or spongy texture, is very great, so that in some

† l. c.—p. 262.

instances it is almost impossible to destroy the odor without the aid of water or chemical re-agents. The numerous examples of this kind, such as musk, castor, the secretion of the odoriferous glands of the pole-cat, assafœtida, etc., are sufficiently known as to require no further comment. It is only to the odor of the exhalations from the human skin, and to the tenacity with which the minute particles—emanating from the respective secretions from which the odor proceeds—must adhere to neighboring objects, that I wish to direct the attention. The most striking example of this kind is afforded by the dog tracing the trail of his master, or that of another animal in hunting. Many of the most remarkable instances are known and authenticated, in which dogs have traced the trail of their masters to great distances, even into neighboring countries, and which evidently show that a portion of the odorous particles contained in the exhalations of the master, must remain suspended in the air near the ground over which the trail passes, or adhere to the ground itself, notwithstanding the currents of air almost constantly present in the open streets or country. Nothing interrupts the trail but running water, which washes the particles away. In this instance, of course, the olfactory sense of the dog, perceiving these particles, is much more acute than that of man; this, however, does not invalidate the argument, as the particles must be there in order to leave an impression upon the dog's olfactory organ. Still more remarkable is the olfactory sense of insects, by whatever organ it may be represented, which attracts them to matters, which in many instances, even, possess no apparent odor, whilst they certainly must emit particles diffusing throughout the atmosphere to considerable distances to come into contact with the organs of special sense of these animals. It is a known fact, and one to which I can testify from experience, that in hot weather the odoriferous trail of a full-blooded negro may be perceived by the human olfactory sense for a considerable distance.

How strong and dense the exhalations of healthy persons even are, may easily be judged from the offensive odor of the air of small and closed sleeping rooms, if entered in the morning before they have been ventilated; and the tenacity with which these exhalations will adhere to linen clothes or other garments, may be judged from the smell of those articles after having hung for some time in rooms overcrowded with people, as met with among the indigent classes of society.

Now, if we consider the amount of exhalation emanating from the skin and lungs of a yellow fever patient during the course of the disease, and moreover characterized by the peculiar odor already referred to, it must be obvious that the atmosphere of the room, even with proper ventilation, must become charged with the poisonous particles contained in the exhalation; and if there are several cases in the same house, the air within must eventually be contaminated with the poison; and attenuated as this poison may be, the particles representing it will surely adhere to surrounding objects, or escape through the open windows to be carried to neighboring houses. And, if we furthermore consider the complex nature of the intercourse kept up by the inhabitants of a large commercial city, like New Orleans, the facility with which a considerable portion of the poison, adhering to the garments of the nearer friends and visitors of a number of patients, will be carried to different places and houses must be easily understood by the philosophically thinking mind. For it cannot be supposed that every man, woman, or child, whose friends are lying sick in their homes with yellow fever, will break off their social and commercial intercourse with the rest of the people. In fact, this intercourse is so complex, and its threads so intricately interwoven, as to render the tracings utterly impossible, even to the sharpest detective, and at the very beginning of an epidemic. The many failures that have occurred in the attempt at tracing the first cases of yellow fever of an epidemic to their true origin are due to this complexity of social and commercial intercourse. But more especially to the fact, that it is almost always the *fatal* cases which first attract the attention of the authorities, while the *milder* ones, not seen by the physician, and perhaps not even recognized or suspected by the patient or his friends, themselves, and constituting the larger number, will get well without any medical aid. But, while the latter may not be noticed, they nevertheless reproduce and spread the noxious poison, as well as those terminating fatally.

In my paper on "The Nature of the Poison of Yellow Fever,"

published in the *New York Medical Journal*, May, 1879, I expressed the idea that the poison of yellow fever, similar to the virulent poison of putrid substances, increased in intensity with each individual through whom it passes. By a subsequent and more mature reflection, however, I have come to the conclusion that experience does not prove this supposition, as, otherwise, the intensity of the poison would soon reach a degree at which it would affect every person without regard to their degree of susceptibility—though it is not impossible that the virulence of the poison emanating from some individuals may be higher in degree than that emitted by others. But, even without this supposition, the quantity of poison emanating from any yellow fever patient, and possessing no greater virulence than that which originally entered his system, is sufficient to explain the spread of the disease.

As regards the transmission of the poison of yellow fever by ships from port to port which is, as mentioned before, a historical fact, it seems to me that even here the poison is generally carried by the garments and effects of the sailors or passengers, though I would not venture to deny the possibility of its being transmitted by the cargo, especially if the articles of which it consists had been exposed to the air of an infected locality. If cases of yellow fever have occurred during the journey, then there remains no doubt that the poison was carried by the clothing and baggage.

Regarding the decline and final disappearance of an epidemic of yellow fever, it is very generally believed that the noxious poison is destroyed by frost. The truth of this supposition, however, is only apparent, as it cannot be proved by any positive and reliable facts. On the contrary, it is a fact that, at New Orleans, the epidemics had almost always nearly disappeared when the first frost occurred, and that frequently new cases arise long after the appearance of this meteorological phenomenon. Thus, in the epidemic of 1867, I paid the first visit to my last case on the tenth day of December, a case which, though not proving fatal, was nevertheless a severe one, accompanied with jaundice and extensive hæmorrhages of the lungs, the patient being a foreigner who had arrived at New Orleans about two months before she was attacked by the disease. The same thing happened, as far as I

remember, at Memphis and other places situated more northerly than New Orleans, where the disease continued to prevail for some time after the appearance of the frost. Thus, the epidemic always reaches its maximum height a considerable time before frost appears, and there remains no doubt that its decline is chiefly owing to the decrease of the number of those persons susceptible to the action of the yellow fever poison, but also *to the lower temperature condensing the latter upon the objects to which it has adhered, and interfering with its evaporation, both from these objects and from the bodies of the affected individuals, and thus diminishing the chances of its being inhaled by other persons.* At the same time, the systems of those persons who thus far escaped the disease are reinvigorated with the approach of cooler weather in the month of October. These are, as I suppose, the chief causes to which the decline of the disease may be attributed, though the germ-theorists firmly believe that the frost kills the living germs, representing, as they suppose, the cause of yellow fever.

And to causes reverse to this may be attributed the re-appearance of the disease, especially in the year succeeding that of an epidemic, for it is very obvious *that during an extensive epidemic a considerable portion of the noxious poison must have remained adherent to clothing and to the walls of rooms, etc., as well as to mattresses and other bedding by which it had been absorbed with the perspiration of the patient,* some of which, after having existed during the winter and spring—perhaps unexposed to the atmosphere and light, and in a state of condensation—*may, with the returning heat of the summer, associated with moisture, be again evaporated and resume its activity.* There remains no doubt that in this manner the yellow fever epidemic, occurring in Memphis in 1879, arose from the clothing and effects of yellow fever patients from the epidemic of 1878, as soon as the shoemaker opened his box, in which they had been kept during the winter and spring. In the same manner arose those cases occurring in New Orleans in 1879, which, besides, might have increased in number to assume the form of an epidemic, if the board of health had not isolated them as far as circumstances would permit. All so-called sporadic cases arise in this or a similar manner, and it is by no means improbable that the noxious poison of yellow fever, like that of small-pox, scarlet fever, measles, and other contagious diseases, may seldom become entirely extinct in places which it is accustomed to haunt, but constantly lurk about in dark corners deprived of fresh air, to resume its activity as soon as exposed to heat and moisture—and that, therefore, its fresh importation by ships is not always a strictly essential condition.

A further proof, however, that cold does not destroy the noxious poison of yellow fever, or of any other contagious disease, may be found in the known fact that low temperatures, on the contrary, preserve all organic substances. And to illustrate this fact we have only to point to the huge cadavers of the mammoth, buried for thousands of years in the ice-fields of Siberia, the flesh of which was so well preserved when discovered by the herdsmen of those icy regions, that they made use of it for feeding their dogs. But even if the cause of yellow fever were represented by living organisms, such as bacteria, it could neither be destroyed or killed by the cold of our winters, nor by a much lower temperature. This has been sufficiently proved by a number of experiments, but most forcibly by *Frisch*,* who exposed bacteria to a temperature as low as 87.5° C., below zero, a condition from which they revived with the thawing of the liquid.

Much has been said and written about certain meteorological conditions required for the activity and propagation of the yellow fever poison, such as a certain amount of moisture in the air together with a continued tropical heat, a deficiency of ozone, etc. Though nothing positive concerning these conditions has thus far been ascertained, I would not deny the probability of such particular conditions; for the fact that yellow fever has assumed the dimensions of extensive epidemics only at shorter or longer periods, while, on the other hand, it has frequently existed it the form of so-called minor epidemics, or been represented only by a few sporadic cases, shows that the same atmospheric conditions are not always present. Neither does the noxious poison,

---

* *Virchow u. Hirsch*, Jahresbericht fuer das Jahr, 1877, Vol. I., p. 285.

after its absorption into the blood, manifest its activity in the same degree in different persons, a phenomenon to be explained by the different degree of susceptibility which these persons possess to the effects of the poison, depending probably upon the particular state of their constitutions at the time when it is brought in contact with the latter. Thus we frequently meet with persons who have been exposed in many different ways to the action of the poison during several epidemics without ever being affected, but who, after the lapse of many years, are finally attacked by the disease. This was the case with myself; for while I escaped taking the disease in former epidemics, I became infected in 1878, while I was engaged in examining the blood of living patients at the Charity Hospital, and when, in order to obtain the specimen of blood, I was obliged to remain for some time in very close contact with the patient, thus inhaling his exhalations. In such cases the disease mostly appears in the milder form. Nothing is known of the nature of this singular immunity, whether it is owing to a peculiar constitution of the blood or of the nervous system at the time of exposure. Nevertheless, it has at times been taken hold of by the "non-contagionists," and forwarded as a proof of the non-contagiousness of yellow fever. Such an argument, however, must fall to the ground in considering that the same phenomenon is observed equally as well in scarlet fever, measles, and small-pox, etc., the contagiousness of which is universally acknowledged. Thus, a number of years ago, I attended a little girl about seven years old, affected with confluent small-pox; it was the most severe case of which I ever had charge, for the eruption extended from the face over the whole scalp, almost forming a mask, and was associated with considerable suppuration and a loss of the entire hair. In this case, remarkable as it may appear, the mother of the patient had during the whole course slept with it in the same bed, and the grandmother in the same room, without either of them contracting the disease, while the father by only once approaching the bed, at the time when the pustules were drying, rapidly caught the disease which fully developed upon him. As far as our knowledge extends at present, we are unable to offer any explanation of these contradictory facts, and it appears to me useless to indulge in empty speculations.

Although the customary assertion that native-born or long acclimated persons enjoy immunity from an attack of yellow fever has now been sufficiently proved as unfounded, it cannot be denied that, in most instances, acclimatization greatly ameliorates the severity of the attack. This is most strikingly observed in unacclimated persons, especially in those who have lived in northern latitudes, beyond the yellow fever zone, and who are generally the first victims to the disease. And it has become a general rule, corroborated by all writers, that the nearer a person has been born and lived to the North Pole, the more liable he is to be attacked by the disease, and the smaller his chances will be for recovery. The fallibility of this assertion, however, has also been shown by our experience during the epidemic of 1878, when the French, Italian, and Spanish population of New Orleans suffered equally as much, *if not more*, from the disease than that of Teutonic or Anglo-Saxon origin; even the Negroes did not remain exempt. Nevertheless, as regards non-acclimated or acclimated persons, it is not improbable that the air of the yellow fever zone may in some essential points differ from that of northern climes, enabling it to exert a certain unknown influence upon the constitution of the blood of those persons who breathe it, adapting it, so to speak, to the yellow fever poison. And to this circumstance it is mainly due that the rate of mortality during the epidemic of 1878 had been proportionately much larger in Memphis, Vicksburg, Holly Springs, and numerous other places, than in New Orleans, or even in other parts of Southern Louisiana. Besides this, the proportion of persons who enjoy an immunity of the disease, by having been previously affected by it, to the whole population, is greater at New Orleans than at the places above mentioned.

### TREATMENT.

Although from the facts relating to the clinical phenomena, pathology, and probable nature of the specific poison of yellow fever, which I have presented to the reader in the preceding sections of this treatise, a rational course of treatment may be easily deducted, some additional remarks will be demanded to direct the attention of the practicing physician to certain apparently minor, but really important points.

The most simple method of arresting a disease, produced in a previously healthy organism by the noxious effects of a specific organic poison, would, of course, consist in the neutralization of the latter, inducing a cessation of the pathological processes—if not advanced too far—and a gradual return of the organism to its normal condition. Unfortunately, however, medical science has as yet not discovered the antidote of the yellow fever poison, and we must, therefore, look for another method of treatment, by which we may counteract the effects of the poison, and, moreover, assist in its elimination from the system with as little damage to the patient as possible—a treatment, which, if based upon rational principles, should correspond to the natural efforts which the organism is observed to make in the elimination of other infectious poisons. Numerous examples of this kind are constantly witnessed in the so-called critical sweats, diarrhœa and other discharges, or in the eruptions of some other contagious diseases; for, it would be difficult to prove that these processes, though pathological in character, do not represent the effort and the means by which the unaided organism chiefly rids itself of the foreign intruder, the morbid poison. In taking a simple case of intermittent fever as an example, we meet with many of the phenomena as are witnessed in the first stage of yellow fever, but which, in this instance, will disappear with the termination of the sweating stage. The most rational inference to be drawn from this fact, therefore, is that the poison, or, at least, *the product resulting from its combination with one or the other constituents of the organism*, is eliminated by a secretory, or rather excretory, process of the glands of the skin, or, perhaps, also by the biliary or urinary secretions. The same phenomenon may be observed in small-pox, when the fever abates with the formation of the vesicles, and ceases as soon as the whole eruption is out. From this assertion, however, it must not be inferred that an excessive secretion of the skin, or of the liver and kidneys, will at once eliminate the poison from the system, but it must be remembered, that every gland can, in its normal condition, only perform a certain fixed amount of labor, proportionate to the quantity of its parenchyma, and that any increase obtained by means of artificial or other abnormal stimulation must be followed by a decrease of the normal amount. Besides, some other points and conditions associated with the febrile process, and incompatible with an excessive secretion, must also be taken into consideration. The secretory functions, therefore, should only be moderately promoted if possible, but never be stimulated to excess. The next object in view is to reduce the elevated temperature to near the normal standard for the special purpose of limiting the disintegration of the albuminous substances, which, as is believed by a number of pathologists, is promoted by an abnormally elevated temperature of the body. The third, and perhaps most important point to be observed, however, is to prevent or to allay every excitement of the nervous system.

In passing a review of the clinical phenomena and the pathological anatomy, we find that in all fatal cases the nervous system takes a most prominent part, and in discussing the general pathology of the disease I have endeavored to clearly demonstrate this fact. And it may safely be pronounced that if the integrity of the nervous organs were not disturbed, the organism, if left to itself, would easily get rid of the poison, and the fatality of the disease be removed. This assertion is fully proved by the thousands of cases which recover without any medical aid. The most dangerous of these nervous disturbances is the hyperæmia of the brain, which, as I have shown, is almost always observed in fatal cases after death. For this reason the physician should never neglect to study the condition of the brain of his patient, from the various nervous phenomena manifesting themselves during the course of the disease, particularly during the febrile stage, and by the proper therapeutic means and applications endeavor to keep the congestion of this organ confined within safe limits. The first condition, essential to the safety of the patient must, therefore, be *the perfect rest of the mind, as well as of the body.*

To secure the necessary rest of the mind for the patient is in many cases quite a difficult task for the attending physician on account of the pernicious custom, especially prevailing among the working classes, of the female friends and neighbors entering the sick-room, and going to and fro for the pretended purpose of inquiring after the condition of the patient, but in reality from idle curiosity. I doubt not that many a case of yellow fever has terminated fatally for the want of the proper rest of the mind, and the proper nursing at the *proper* time. It usually happens that when there are too many friends the patient is neglected, and will have to pay the cost with his life. For this reason, the physician, after having taken the case in charge, should at once select one or two of the most intelligent and trusty friends, who voluntarily offer, to stay alternately with the patient, day and night, at least until the crisis is over. Much has been said about professional yellow fever nurses. I have never had a great deal of faith in these persons, as they are strangers to the patient, and frequently too meddlesome, imagining that they know better than the physician, and not carrying out his orders very strictly—not infrequently even supplanting them by their own notions. The most reliable nurses are always the nearer relations and friends of the patient, whose sentiments and interests are interwoven with his own. The impression made upon the patient, by seeing strange faces around him, can neither be favorable. In the epidemic of 1867, particularly, I observed the marked importance of conscientious and careful nursing in a number of severe cases, who, attentively nursed by their nearer friends and relations, recovered, while others, surrounded and nursed by strangers, succumbed.

As soon as the first symptoms of the disease appear, therefore, the patient should be confined to bed and made as comfortable as possible, both in body and mind. The physician should contribute to keep up his moral courage by representing to him his case as light as possible, and by explaining the importance of the rest of his mind. Intelligent patients I have usually advised to keep from using their mind at all, but merely vegetate, so to say, in trying to sleep, and to leave all cares to their friends. Regarding the sickroom, it is of the greatest importance that it should be well ventilated, as otherwise, not only the patient, but also those persons who nurse him, will be obliged to breathe his cutaneous exhalations containing the infectious poison. At the same time, however, every direct current of air upon the patient should be scrupulously avoided; he should be lightly covered, but his surface not wantonly exposed.

In discussing the general pathology of the disease, I referred to the probable temporary increase of the biliary functions in the commencement of the disease, and, for this reason, the customary initiation of the treatment by the administration of a mild cathartic, for the purpose of unloading the portal circulation, must be recommended. There are a number of medicines which will answer this purpose. The most popular one in this city, both with physicians and the people, is castor-oil, and, if not rejected by the stomach, answers as well as any other on account of its quick action. A number of physicians administer a dose of calomel, combined with one or the other cathartic, such as jalap, etc. As the calomel produces to a certain extent a bilious stool, this remedy rather appears favorable, especially as we know from the autopsies that in almost all fatal cases the gall-bladder contained a dark brown, tar-like bile. The citrate of magnesia may also be used, though it is frequently rejected by the stomach. Subsequently, in the course of the disease, however, it is better to use an enema instead of the cathartic medicine.

The almost universal custom of giving a hot stimulating foot-bath for the purpose of inviting the blood from the brain to the feet, also, may be of service in the beginning of the disease, but with the necessary caution to be administered after the action of the cathartic, allowing the patient to remain undisturbed afterward. The foot-bath, however, will only prove of advantage in the beginning of the disease.

In order to promote the perspiration, it is customary—at New Orleans—to administer an infusion of orange leaves. These leaves, if possessing diaphoretic properties at all, possess them only in an inferior degree. They are simply used in Louisiana, because they are easily obtained. In the Northern States they would be replaced by "boneset," (Eupatorium perfoliatum), and in Germany by the flowers of "chamomile." And as such infusion, in order to produce a diaphoretic effect, should be administered while hot, but which, in most cases, is neglected, the benefit derived from the orange leaves is only imaginary; even if the cold infusion is taken with the view of allaying the irritation of the stomach. Besides, the administration of hot infusions at a time when the patient suffers already from the excess of heat, both from the weather and the disease, is hardly practicable, as it really contributes nothing to the comfort of the patient. Perhaps one of the most efficient and harmless remedies is the "neutral mixture" with the addition of a little ($\frac{1}{16}$ part) sweet spirits of niter, given in such doses as to keep the skin only in an evenly moist condition, which, however, may be discontinued while the skin remains moist, but resumed as soon as the perspiration ceases, and the skin becomes again dry. This mixture is tolerably well borne by the stomach, whilst the spirit of niter, besides, may slightly stimulate the kidneys. But as with the rise of the fever the thirst of the patient rapidly increases, he finally also refuses this remedy, and demands ice-water, which, if given in proper doses, appears to suit the occasion as well as any article the apothecary could furnish. Unfortunately, however, the thirst of the patient is so great that he loses his self-control, and will fill his stomach with the cold liquid if not interfered with by his nurse; then, small fragments of ice suffered to slowly dissolve in the mouth may answer the purpose.

[TO BE CONTINUED.]

